# Exogenous Gibberellic Acid (GA_3_) and Benzylaminopurine Enhance the Antioxidant Properties of *Vaccinium corymbosum* L. ‘Biloxi’ Fruits Without Affecting Yield

**DOI:** 10.3390/ijms26167984

**Published:** 2025-08-19

**Authors:** Larissa Silva Rodrigues, Caroline Pardine Cardoso, Edson Tadashi Savazaki, Stephane Catarine Rosa Kim, Carolina Ovile Mimi, Iván De-la-Cruz-Chacón, Gisela Ferreira

**Affiliations:** 1 Department of Biodiversity and Bioestatistics, Institute of Biosciences, São Paulo State University (UNESP), Botucatu. Prof. Dr. Antonio Celso Wagner Zanin Street, 250, Botucatu 18618-689, São Paulo, Brazil; larissa.s.rodrigues@unesp.br (L.S.R.); caroline.pardine@unesp.br (C.P.C.); stephane.kim@unesp.br (S.C.R.K.); gisela.ferreira@unesp.br (G.F.); 2 Secretary of Agriculture and Supply of the State of São Paulo (SAA/SP), Coordination of Technical and Integral Assistance (CATI), Avenida Nove de Julho Street, 467, Guaiçara 16430-037, São Paulo, Brazil; edson.savazaki@sp.gov.br; 3 Laboratorio de Fisiología y Química Vegetal, Instituto de Ciencias Biológicas, Universidad de Ciencias y Artes de Chiapas (UNICACH), Libramiento Norte Poniente, 1150. Col. Lajas Maciel, Tuxtla Gutiérrez 29039, Chiapas, Mexico; ivan.cruz@unicach.mx

**Keywords:** plant growth regulators, blueberry, phenolic compounds, antioxidant activity, flavonoid fruits, anthocyanin fruits

## Abstract

*Vaccinium corymbosum* L. ‘Biloxi’ is a cultivated blueberry variety valued for its rich content of phenolic compounds, which contribute to its strong antioxidant activity and recognized health benefits. There is little information on the effects of GA_3_ and BA on blueberry, especially when used in combination. This study aimed to evaluate whether GA_3_ and BA alter the yield and quality of *V. corymbosum* ‘Biloxi’ fruits. The experiment included 12 treatments consisting of GA_3_ (25, 50 and 100 mg L^−1^) and BA concentrations (50 and 100 mg L^−1^) alone and combined and a control. The following parameters were analyzed: yield (g) and number of fruits per plant, mass, diameter, pH, soluble solids (SS), titratable acidity (TA), soluble sugars, total phenols, flavonoids, anthocyanins and antioxidant activity. The results indicate that foliar GA_3_ and BA application improved the antioxidant capacity and biochemical composition of fruits, without negatively affecting production traits such as yield, fruit size or maturation period. The increases in antioxidant activity, phenol metabolites (total phenols, anthocyanins and flavonoids), soluble sugars, SS and SS/TA ratio were higher with the combination of GA_3_ and BA at 100 mg L^−1^. These results suggest that the combination of GA_3_ and BA is a promising approach to sustainably improve fruit quality in commercial blueberry cultivation, providing both economic and nutritional benefits.

## 1. Introduction

Blueberry (*Vaccinium corymbosum* L.) is a fruit plant known worldwide for the high antioxidant capacity of its fruits, attributed to the presence of specialized phenolic metabolites, mainly flavonoids and anthocyanins [1,2]. Blueberries are considered one of the five healthiest foods for human consumption [3], and their consumption has been associated with health benefits, including anti-cancer [4], anti-inflammatory [5] and anti-obesity effects [6] and the prevention of cardiovascular diseases [7] and diabetes [8].

The growing global public health awareness of functional foods with multiple benefits has made blueberries popular, causing their consumption to increase significantly [9,10,11]. In this context, contemporary studies on blueberries have been focused on strategies to increase fruit yield and quality [12,13,14,15], including nutraceutical composition (specialized antioxidant metabolites) [16] and visual and sensory patterns related to the physicochemical characteristics of fruits such as mass, diameter, concentrations of sugars and soluble solids and titratable acidity [17].

The knowledge of the physiological effects of plant growth regulators on fruit set can be used to increase fruit yield and quality, as they modulate physiological processes that influence fruit growth and retention, in addition to correcting physiological disorders to improve fruit yield and quality [18].

Gibberellic acid (GA_3_) is a plant growth regulator widely used to act on fruit development, promoting cell division, increasing size and yield and increasing the concentrations of specialized metabolites [19,20,21]. Previous studies indicate that the supply of GA_3_ at the beginning of fruit set in *Vitis vinifera* increased fruit mass and size [22], with enhanced effect when used in combination with synthetic cytokinin forchlorfenuron [23]. Furthermore, GA_3_ application at full bloom increased soluble solids and sugars in *Pyrus communis* fruits [24] and increased the antioxidant activity and the concentrations of total phenols and flavonoids in *Rubus* spp. fruits when used at full bloom and fruit set [25].

Cytokinins are known for their effects on the fruit quality of fruit species, increasing yield, fruit size and concentrations of primary and specialized metabolites when used alone or in combination with other plant regulators [26,27]. In *Malus domestica*, exogenous treatment at full bloom with cytokinin benzylaminopurine (BA) alone or combined with gibberellin GA_4+7_ resulted in fruits with greater mass and diameter [28]. In *Fragaria vesca*, exogenous BA supply promoted the accumulation of sugars, soluble solids, total phenols, flavonoids and anthocyanins in fruits [29].

In blueberry (*Vaccinium corymbosum*), studies have shown the effect of exogenous GA_3_ and BA supply on the yield and physicochemical characteristics of fruits, with variable responses. The application of 100 mg L^−1^ of BA at petal fall increased yield per plant and the average fruit mass; however, the application of 50 mg L^−1^ of this plant regulator and the application of 200 mg L^−1^ of GA_3_ did not change yield per plant or fruit size [30,31]. Regarding the chemical characteristics, the application of 30 mg L^−1^ of GA_3_ at the beginning of fruit development caused an increase in soluble solids [32]. In contrast, there was a reduction in the soluble solids content of fruits when 100 and 150 mg L^−1^ of GA_3_ were exogenously supplied at flowering [33].

When considering the effects of GA_3_ and BA on the antioxidant composition of *V. corymbosum*, a small amount of information is available in the literature and describes their isolated use, demonstrating an increase in the concentrations of flavonoids and anthocyanins with the use of 20 mg L^−1^ of BA [34]. When considering the combined effects of GA_3_ and BA on *V. corymbosum*, no studies were found to date regarding yield, physicochemical characteristics or antioxidant composition. However, in another blueberry species (*V. ashei*), three GA_3_ applications at 500 mg L^−1^ resulted in fruits with greater mass and diameter and increased antioxidant activity [35].

Based on the above, the aim of this study was to evaluate whether plant growth regulators GA_3_ and BA, supplied alone or in combination, alter the yield, physicochemical quality and antioxidant composition of *V. corymbosum* ‘Biloxi’ fruits.

## 2. Results

### 2.1. Productive and Physical Characteristics of Fruits

**Figure 1:**
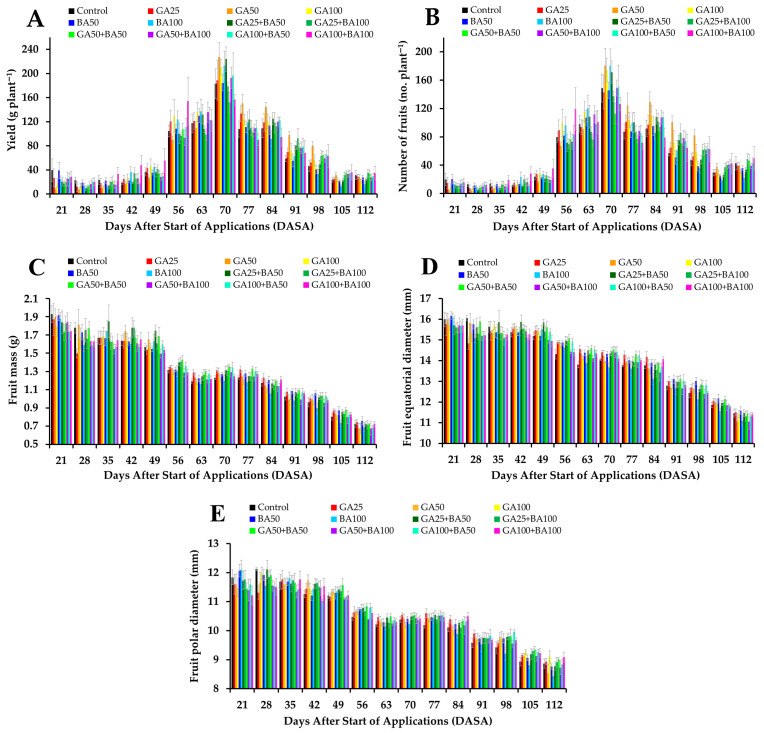
Effect of gibberellic acid (GA_3_) and benzylaminopurine (BA) on yield (g plant^−1^) (**A**), number of fruits (no. plant^−1^) (**B**), fruit mass (g) (**C**), fruit equatorial diameter (mm) (**D**) and fruit polar diameter (mm) (**E**) in *Vaccinium corymbosum* ‘Biloxi’ fruits in the days after the start of application (DASA) in the 2023 yield cycle. Bars represent the standard error of the mean.

Throughout the 14 collections, yield (g plant^−1^) and number of fruits (no. plant^−1^) showed similar patterns in treatments with plant growth regulators and control (Table S1—Supplementary Material). These variables increased until 70 days after the start of application (DASA), characterized as peak yield, which subsequently decreased until the end of the yield cycle (128 DASA) (Figure 1A,B).

Fruit mass (g) and fruit equatorial and polar diameters (mm) also showed similar patterns between treatments with plant growth regulators and control (Table S1). However, these variables were reduced throughout the period (Figure 1C–E).

Therefore, it could be inferred that the supply of GA_3_ and BA, alone or in combination with two applications carried out at the reproductive stage of plants on 28 July and 11 August 2023, was not sufficient to increase fruit yield and number of fruits per plant, as well as the mass and fruit equatorial and polar diameters by any of the 14 collection dates evaluated. Likewise, no variations were observed in the mean values of these variables considering the yield cycle as a whole (cycle mean) (Table S2—Supplementary Material).

### 2.2. Antioxidant Composition of Fruits

At peak production (70 DASA), the combination of GA_3_ with BA at their highest concentrations (GA100 + BA100) promoted a simultaneous increase in antioxidant activity and concentrations of total phenols, flavonoids and anthocyanins in fruits (Table 1). Similar responses were observed with the use of GA100 + BA50, except that this combination did not increase the concentration of flavonoids. These results demonstrate the combined action of plant regulators, unlike when they were used alone, for example, GA100 promoted an increase in the concentration of anthocyanins and antioxidant activity, while BA100 increased flavonoids and reduced antioxidant activity (Table 1).

Plants treated with the highest GA_3_ concentrations (GA50 and GA100) also produced fruits with higher antioxidant activity, with increases in flavonoids (GA50) and anthocyanins (GA100). However, the antioxidant activity of fruits was reduced when plants received the lowest GA_3_ concentration (25 mg L^−1^) and when this concentration was associated with the lowest BA concentration (GA25 + BA50), similar to results already observed with BA100 (Table 2).

Based on the results, GA100 + BA100, GA100 + BA50, GA25 + BA50, GA100, BA100 treatments were selected to be analyzed at a date closer to the supply of plant growth regulators, at the beginning of the yield cycle (28 DASA) and at the end of the yield cycle (112 DASA), with the aim of analyzing the intensity of the effect of GA_3_ and BA more broadly throughout the yield cycle.

At the beginning of the yield cycle (28 DASA), as well as at peak yield, plant growth regulators GA_3_ and BA (isolated and combined) had a significant effect on the specialized metabolites and/or antioxidant activity of fruits (Table 1 and Table 2). However, at the end of the yield cycle, the results did not demonstrate significance (Table S3).

When considering the results at peak yield and at the beginning of the yield cycle, the significant effects of the GA_3_ and BA combination at their highest concentrations (GA100 + BA100) are confirmed. This was the only treatment that provided an increase in the antioxidant activity of fruits and an increase in the concentrations of total phenols, flavonoids and anthocyanins, except at the beginning of the cycle, when no changes in the concentration of flavonoids were detected (Table 1 and Table 2).

Although the other treatments showed an increase in at least two specialized metabolites, the antioxidant activity of fruits remained unchanged (Table 2). These responses differ from those found at peak yield, where the antioxidant activity increased with GA_3_ at the highest concentration (GA100) and decreased with BA100 and GA25 + BA50 treatments (Table 1).

### 2.3. Chemical Characteristics of Fruits

In addition to the antioxidant composition of fruits, at peak yield (70 DASA), plant growth regulators also promoted variations in the concentrations of soluble sugars, SS, TA and SS/TA ratio, without, however, modifying pH (Table 3).

The concentrations of soluble sugars in fruits increased with the use of all combinations of plant growth regulators and the highest GA_3_ and BA concentrations alone (100 mg L^−1^). Similar results were found for SS, except for combinations in which GA_3_ was applied at lower concentrations (GA25 + BA50 and GA25 + BA100), where SS resembled control (Table 3).

GA100 + BA100, GA50 + BA50 combinations and the highest GA_3_ and BA concentrations resulted in fruits with higher concentrations of soluble sugars and SS, without altering TA. A significant increase in the titratable acidity of fruits was observed with BA at 50 mg L^−1^ and when this concentration was combined with 100 mg L^−1^ of GA_3_; this was similar to when 100 mg L^−1^ of BA was combined with 25 and 50 mg L^−1^ of GA_3_ (Table 3).

The SST/AT ratio values of fruits were not altered with the use of the highest GA_3_ and BA concentrations isolated and combined (GA100 + BA100), nor with the GA25 + BA50, GA50 + BA50 combinations, compared to control, while the other treatments caused reductions in these values (Table 4).

The chemical characteristics of fruits from plants treated with GA100 + BA100, GA100 + BA50, GA25 + BA50, GA100, BA100 were analyzed at the beginning of the cycle (28 DASA) and at the end of the yield cycle (112 DASA), similar to procedures performed for the antioxidant composition.

As at peak yield, at the beginning of the yield cycle, plant growth regulators promoted variations only in the concentrations of soluble sugars, SS, TA and SS/TA ratio of fruits (Table 3 and Table 4). The increase in soluble sugars was also detected with all GA_3_ and BA treatments, alone and in combination. In addition, similar results were found for SS, except with GA100, which at the beginning of the yield cycle resulted in fruits with SS concentration similar to control (Table 3 and Table 4).

The GA100 + BA100 combination stimulated the increase in the concentrations of soluble sugars and SS without causing changes in TA, as previously reported at peak yield (Table 3 and Table 4). Furthermore, this treatment was the only one to cause an increase in the SS/TA ratio values of fruits.

Similar responses to peak yield were also observed with the GA100 + BA50 combination at the beginning of the yield cycle, demonstrating a simultaneous increase in soluble sugars, SS and TA (Table 3 and Table 4). Furthermore, this was the only treatment that caused changes in the characteristics of fruits at the end of the yield cycle, showing increase in soluble sugars and SS (Table S4).

Treatments with GA25 + BA50 and with GA_3_ and BA alone (100 mg L^−1^) increased TA concentrations at the beginning of the yield cycle, differing from responses found at peak yield (Table 3 and Table 4).

In summary, when analyzing the antioxidant composition and chemical characteristics of fruits on the three collection dates evaluated, a greater influence of plant growth regulators was observed on dates closer to application (beginning of the yield cycle and at peak yield) than at the end of the yield cycle.

## 3. Discussion

### 3.1. Productive and Physical Characteristics of Fruits

There is no unanimity in the literature regarding the role of GA_3_ and BA in the development of blueberries (*V. corymbosum*). Thus, the fact that GA_3_ and BA did not significantly stimulate the increase in fruit yield and number of fruits per plant, fruit mass and fruit equatorial and polar diameters, either in individual collections or in the average of the yield cycle, is supported by other studies, both with GA_3_ [31,36] and with BA [30].

In the study by Milić et al. [31], the single GA_3_ application of 200 mg L^−1^ in *V. corymbosum* ‘Duke’ and ‘Bluecrop’ fruits did not alter yield per plant, mass and fruit equatorial diameter in the average of the yield cycle, carried out at petal fall, similar to this study with GA_3_ application of 25, 50 and 100 mg L^−1^. Koron and Stopar [30] demonstrated that the BA application of 50 mg L^−1^ at petal fall did not alter yield per plant, mass and fruit equatorial and polar diameters of *V. corymbosum* ‘Bluecrop’ fruits, as observed with BA at 50 and 100 mg L^−1^ for the ‘Biloxi’ cultivar in this study. In contrast, there are studies that demonstrated increases in the productive and physical characteristics of *V. corymbosum* fruits with the use of BA [17,31].

Variations in responses occur because the effect of plant growth regulators may vary depending on concentration, number and time of applications [37], genotypes evaluated [38], plant phenological stage at the time of treatment [39] and environmental conditions [40]. In this context, in the present study, *V. corymbosum* ‘Biloxi’ plants received two GA_3_ applications at concentrations of 25, 50 and 100 mg L^−1^ with an interval of 14 days, when they had floral organs and fruits. Zang et al. [35] observed increase in fruit mass and equatorial and polar diameters of ‘Powder blue’, ‘Garden blue’ and ‘Climax’ fruits with three GA_3_ applications at concentration of 500 mg L^−1^ with an interval of 5 days and prior to the beginning of flowering; therefore, the phenological stage may explain the differences in results. Similarly, Wang et al. [41] also observed an increase in the mass of blueberry ‘Baldwin’ fruits with GA_3_ supply of 50 and 100 mg L^−1^; a result different from that of the present study, since the flowering branches were immersed in GA_3_ solutions for five seconds twice, with an interval of 5 days.

Regarding the use of BA, Simpson et al. [17] observed an increase in the mass and diameter of blueberry ‘OB1’ fruits with the supply of BA at the same phenological stage (presence of floral organs and fruits) compared to this experiment; however, the application conditions were different, two applications of 150 mg L^−1^ with an interval of 27 days with average local temperature between 13 °C and 23 °C, different from the conditions evaluated in this study (two BA applications of 50 and 100 mg L^−1^ with an interval of 14 days with average local temperature between 20 °C and 23 °C), which may indicate that higher concentrations can stimulate yield. Similar to the effect of phenology with GA_3_, Milić et al. [31] found that the BA application of 100 mg L^−1^ at petal fall in *V. corymbosum* ‘Duke’ and ‘Bluecrop’ fruits demonstrated an increase in fruit yield and mass; however, the experiment was carried out in colder environmental conditions in Serbia, different from conditions in southeastern Brazil.

The fact that GA_3_ and BA applications did not affect fruit production and physical characteristics can be explained by the interaction between promoter and inhibitory plant growth regulators [31,37]. Exogenous applications modulate hormonal balance by stimulating or inhibiting the synthesis of the plant’s internal phytoregulators. These interactions can be synergistic, where two or more hormones work together to amplify an effect, or antagonistic, where one hormone counteracts the effect of another, thus altering growth patterns and developmental processes [42]. For example, BA and forchlorfenuron are known to increase fruit mass by stimulating cell division [43,44]; in particular, forchlorfenuron increased berry size and fruit set in *V. corymbosum*. Meanwhile, GA_3_, commonly used to achieve higher yields and fruit set in *Vaccinium* spp., sometimes reduces berry size and delays harvest [45]. That is, the interaction between exogenous applications and endogenous concentrations of plant growth regulators and inhibitors may have nullified the effect on fruit production [42], without demeriting it.

The low plant sensitivity to exogenous application at certain concentrations and growth stages is also important. GA_3_ produces inconsistent results depending on concentration, number of applications and time of application [37]. For example, triple GA_3_ application of 500 mg L^−1^ to *V. ashei* blueberries, 30 days after harvest, increased the return of flowering, vegetative growth and fruit size in three cultivars [35]; while the only post-flowering GA_3_ application of 200 mg L^−1^ to *V. corymbosum* had no stimulating effect on either yield or average fruit size and also no effects on fruit mass in individual harvests, fruit set and vegetative growth in several cultivars [31].

### 3.2. Chemical and Antioxidant Characteristics of Fruits

In contrast to the lack of responses in the increase in the productive and physical characteristics of fruits, the novelty of the synergistic action of GA_3_ and BA in increasing the concentrations of total phenols, flavonoids, anthocyanins and antioxidant activity, in addition to increasing sugars, SS and SS/TA ratio in blueberry ‘Biloxi’ fruits, is highlighted.

The high antioxidant activity of blueberries is positively correlated with their composition, especially phenols, flavonoids and anthocyanins [46,47,48,49], which act directly in the elimination of free radicals present in the human body, resulting in numerous health benefits [50,51].

In previous studies with blueberry plants, plant growth regulators were used alone, demonstrating an increase in the concentrations of flavonoids and anthocyanins in *V. corymbosum* fruits with the use of 20 mg L^−1^ of BA [34]. Regarding the use of BA in this experiment, at peak yield, the highest concentration used (100 mg L^−1^) reduced the antioxidant activity of fruits, while the lowest concentration (50 mg L^−1^) did not alter the antioxidant activity but increased flavonoids and anthocyanins, similar to the study by Pérez-León et al. [34], also in *V. corymbosum* ‘Biloxi’ fruits.

It was found that GA_3_ (50 and 100 mg L^−1^) increased the antioxidant activity of fruits at peak yield; however, lower values compared to control were obtained when the lowest concentration (25 mg L^−1^) was used, which indicates the need for higher GA_3_ concentrations to stimulate antioxidant activity.

The explanations for the action of plant regulators in regulating the antioxidant composition of fruits lie in changes in physiological and biochemical metabolism and in the expression of structural genes [52].

According to Montero et al. [53], GA_3_ application increases the activity of enzymes phenylalanine ammonia-lyase (PAL) and tyrosine ammonia-lyase (TAL), involved in the biosynthesis of phenolic compounds, which results in higher concentration of anthocyanins, which was observed in *Fragaria* × *ananassa* fruits. Similarly, an increase in the activity of the PAL enzyme was also obtained with the use of BA, which resulted in higher concentrations of phenolic compounds in *V. corymbosum* [34] and *Litchi chinensis* [54]. Considering that GA_3_ and BA are involved in the activity of PAL and consequently increase the biosynthesis of phenolic compounds, it could be inferred that the association of these regulators was responsible for the increases in the concentrations of total phenols, flavonoids and anthocyanins observed with the combinations used in this study, especially GA100 + BA100, GA100 + BA50 and GA50 + BA100.

Regarding gene regulation, there are reports that, in *Vitis vinifera*, treatment with GA_3_ regulated the expression of genes related to the biosynthesis of total phenols, flavonoids and anthocyanins (*PAL7*, *4CL*, *C4H*, *CHS*, *CHI*, *F3′H*, *F3H* and *LDOX*), promoting the increase in these compounds in fruits [19]. Treatment with BA in *Fragaria vesca* also positively regulated the expression of genes related to the biosynthesis of anthocyanins (*PAL6*, *CHS*, *MYB1* and *MYB10*), increasing their concentration in fruits [29]. Although gene expression was not studied in this research, this information suggests that this mechanism was activated with the most effective GA_3_ and BA concentrations used in this study.

Similar responses with exogenous GA_3_ and BA application in the synthesis of phenolic compounds and antioxidant activity were also found in other fruit species. Exogenous GA_3_ resulted in *Malus domestica* and *Rubus* spp. fruits with high antioxidant activity and higher concentrations of total phenols and flavonoids [25,55] and provided an increase in the concentration of anthocyanins in *Fragaria* × *ananassa* [56] and *Vitis vinifera* fruits [57,58], while treatment with BA caused an increase in the concentrations of total phenols and flavonoids in *Fragaria* × *ananassa* fruits [59] and an increase in the concentrations anthocyanins in *Prunus domestica* fruits [60].

Another aspect to be analyzed is that the increase in the concentrations of soluble sugars obtained by the action of exogenous GA_3_ and BA may have helped to increase the antioxidant composition of blueberry ‘Biloxi’ fruits, considering that these sugars can be directed to the synthesis of phenolic compounds [61].

With the use of GA_3_ at its highest concentration (100 mg L^−1^), an increase in total sugars, anthocyanins and antioxidant activity was observed, similar to that observed in *Vitis vinifera*, whose increase in sugar concentrations in fruits caused by treatment with GA_3_ promoted the synthesis and accumulation of anthocyanins [19]. In addition, the concentration of anthocyanins was also increased with the use of various combinations (GA100 + BA100, GA100 + BA50 and GA50 + BA100) as well as sugars, while maintaining high antioxidant activity. A study carried out in *Eucalyptus* suggests that exogenous BA can alter the metabolism of sugars and, thus, activate the components of the flavonoid synthesis pathway in plants, facilitating the accumulation of these compounds [62], which seems to have occurred in this experiment with BA at 100 mg L^−1^; however, the antioxidant activity was reduced at peak yield.

The concentrations of sugars, SS, acids and the SS-to-acids ratio are common indicators of blueberry quality, directly influencing flavor [63]. In this context, the responses in this experiment for SS and soluble sugars with the highest GA_3_ and BA concentrations and the various combinations that resulted in sweeter fruits are supported by the study by Sun et al. [32], in which increase in SS was also found in *V. corymbosum* ‘Bluecrop’ fruits, but only with GA_3_ application of 30 mg L^−1^ at the beginning of fruit development. In contrast, Hu et al. [33] found reduction in SS in *V. corymbosum* ‘Sharpblue’ fruits with GA_3_ at 100 and 150 mg L^−1^. However, the flowering branches were immersed in GA_3_ solutions for five seconds twice, with an interval of 5 days, and this distinct application may explain the differences found.

Similar responses regarding the increase in sugar and SS concentrations in fruits were also found in other fruit trees, such as *Pyrus communis* [24], *Mangifera indica* [64] and *V. vinífera* [19,65] treated with exogenous GA_3_, in *Fragaria vesca* after treatment with BA alone [29] and in *Prunus avium* when BA was combined or not with gibberellin GA_4+7_ [66].

The increase in sugars and SS in fruits due to the action of GA_3_ is attributed to the increase in differentially expressed genes related to starch and sucrose metabolism, making the conversion of these carbohydrates into soluble sugars faster [67], and also the ability to promote the rapid mobilization of photosynthetic metabolites from other parts of the plant towards fruits, resulting in greater translocation and accumulation of sugars in ripe fruits [68,69].

Regarding cytokinin, it has been demonstrated that exogenous treatment regulates the metabolism of endogenous cytokinin in fruits, contributing to increasing the concentration of sugars in the final development stage [70]. This occurs because cytokinin has the potential to increase the absorption capacity of fruits by promoting cell multiplication or sustaining absorption activity through the regulation of sucrolytic enzymes, allowing greater acquisition of photoassimilates [68]. In addition, cytokinins can induce greater activity of sugar transporters and invertases [71].

The TA of fruits also underwent changes at the beginning of the yield cycle and at peak yield, demonstrating a significant increase when some GA_3_ and BA concentrations were used alone or in combination. Regarding the use of GA_3_ alone, Zang et al. [35] demonstrated that three GA_3_ applications of 500 mg L^−1^ performed prior to the beginning of flowering in *V. ashei* ‘Powder blue’ fruits resulted in reduction in fruit TA, unlike what was observed in this study. With BA, Pérez-León et al. [34] also observed a reduction in fruit TA when eight BA applications of 10 mg L^−1^ were performed during the vegetative development of *V. corymbosum* ‘Biloxi’ fruits. In this context, the different results found can be attributed to the differences in concentrations used and the phenological stage of plants at the time of application.

Similar responses regarding the increase in fruit TA through exogenous GA_3_ were found in *V. vinifera* [72] which can be explained by the fact that GA_3_ promotes the expression of genes associated with the synthesis of organic acids, increasing their concentration [67]. Additionally, BA used alone in *P. avium* [66] or combined with GA_3_ in *M. indica* [73] also resulted in increased fruit acidity due to a possible delay in ripening. Despite the above, the acidity values found corroborate the standard established for the quality of blueberry fruits, with values ranging from 0.3 to 1.3%, based on citric acid [74].

Treatments with plant growth regulators did not cause changes in the pH of fruits at any of the dates evaluated, which was also observed in *V. vinifera* fruits treated with GA_3_ [75] and in *Prunus armeniaca* fruits using GA_3_ and benzylaminopurine alone [76] and, also, when benzylaminopurine was combined with gibberellin GA_4+7_ [77]. It is noteworthy that the pH values obtained are close to the average value established in the literature for blueberry ‘Biloxi’ fruits, which is equivalent to 3.2 [78].

Another point to be highlighted, regarding this study, is the lower effect observed by the action of GA_3_ and BA on the chemical characteristics and antioxidant composition of fruits collected at the end of the yield cycle. In this context, Zang et al. [35] reported that the effect of GA_3_ on maintaining the quality of *V. ashei* fruits was greater on the first dates of fruit collection in relation to the end of the yield cycle, which indicates the need for more applications in order to maintain the highest fruit quality throughout the cycle.

## 4. Materials and Methods

### 4.1. Site Characterization

The study was carried out from July to November 2023 in a commercial cultivation of *V. corymbosum* ‘Biloxi’ fruits located in the municipality of Pongaí, state of São Paulo, southeastern Brazil, with geographic coordinates of 21°47′35″ S, 49°22′31″ W and altitude of 458 m a.s.l. The soil of the experimental area is classified as red yellow argisol with sandy/medium texture and gently undulating relief [79].

The climate of the region, according to the Köppen-Geiger classification, is of the Aw type, characterized as tropical with dry season, with dry winter and rainy summer [80]. Meteorological data during the experimental period were monitored daily (Table 5) using a datalogger (Instrutherm, São Paulo, Brazil, model HT900) and a rain gauge (Incoterm, Paris, France, model 4755).

### 4.2. Plant Material

‘Biloxi’ blueberry plants were three years old and originated from micropropagation. The crop was established in the soil in furrows of 60 cm in width and 60 cm in depth filled with substrate of dried natural rice husk (without burning) and non-composted sawdust at ratio of 3:1, with spacing of 3 m between furrows and 0.5 m between plants.

Irrigation and mineral nutrition were carried out by means of a drip system, aiming to provide per cycle 660 L of water per plant and N: 200 kg ha^−1^; P_2_O_5_: 70 kg ha^−1^; K_2_O: 220 kg ha^−1^; Ca^2+^: 80 kg ha^−1^; Mg^2+^: 40 kg ha^−1^; SO_4_^2−^: 250 kg ha^−1^; Zn^2+^: 20 kg ha^−1^; Fe^2+^: 500 g ha^−1^.

To conduct the study, plants similar in height (75 cm), canopy diameter (90 cm), vigor and health were selected, standardized with twenty lateral branches.

### 4.3. Experimental Design and Treatments

A randomized block design was adopted, with twelve treatments (Table 6), five replicates and three plants per plot. Plots were separated by at least one border plant and the experimental area was delimited so as to present one border plant of at least 3 m in width on its perimeter.

Treatments consisted of gibberellic acid (ProGibb^®^, Kansas City, MO, USA, 400 g of GA_3_ per kg of the commercial product, Sumitomo Chemical) and benzylaminopurine concentrations (MaxCel^®^, Kowloon, Hong Kong, China, 20 g of BA per liter of the commercial product, Sumitomo Chemical), alone and combined, in addition to control without plant regulators. Silwet^®^ (Momentive Performance Materials Inc., Garrett, IN, USA) at a concentration of 0.05% was used as surfactant in all treatments.

Treatments were applied twice, with an interval of 14 days (28 July and 11 August 2023). On 28 July, plants contained 76.87% floral organs (swollen buds, open buds, open flowers and fallen petals) and 23.13% green fruits of variable size, while on 11 August, they presented 60.92% floral organs, 37.55% green fruits and 1.53% fruits changing color.

Applications were performed by means of foliar spraying in the morning (starting at 06:00 a.m. and ending at 10:00 a.m.) with 1.0 L of solution per treatment, throughout the plot, using a properly calibrated electric sprayer (Brudden, Pompéia, Brazil, Practical model). To avoid contamination between plots, a protective cover was used to isolate plants and, after each treatment, the sprayer was washed three times.

### 4.4. Evaluations

Ripe fruits from each treatment replicate were manually collected at weekly intervals during the 91-day period, totaling 14 collections. Collections began with the emergence of the first ripe fruits, on 18 August 2023, 21 days after the start of application (DASA), and ended on 17 November 2023, 112 DASA. Data regarding the productive and physical characteristics of fruits were obtained in each collection.

#### 4.4.1. Productive and Physical Characteristics of Fruits

- Yield (g plant^−1^): all fruits collected from the three plants of each treatment replicate were weighed on an electronic scale with accuracy of 0.01 g. Subsequently, the average was calculated to determine yield per plant.
- Number of fruits (no. plant^−1^): all fruits collected from the three plants of each treatment replicate were counted. Subsequently, the average was calculated to determine the number of fruits per plant.
- Fruit mass (g): obtained through the quotient between yield and number of fruits per plant.
- Fruit equatorial diameter and polar diameter (mm): obtained by the average of 20 fruits randomly collected per replicate individually measured with a digital caliper with precision of 0.01 mm (Mitutoyo, Neuss, Germany, model 500-196-30).

The statistical analysis of data was individually performed on each collection date, considering the yield cycle as a whole, based on the total average data of yield and number of fruits and the average data of mass and fruit diameter, referring to the 14 dates.

#### 4.4.2. Chemical and Antioxidant Characteristics of Fruits

In each collection, samples of a maximum of 60 ripe fruits from each treatment replicate were stored on the same day in a freezer at −20 °C until analysis.

From the 14 collection dates, peak yield was detected for analysis of pH, soluble solids (SS), titratable acidity (TA), soluble sugars, total phenols, flavonoids, anthocyanins and antioxidant activity.

At this time, half the number of fruits from each sample (30 fruits) were removed and thawed at room temperature for 15 min [81]. Fruits were macerated and homogenized with a mortar and pestle and filtered with sterile gauze to obtain the concentrated juice, which was used for the following analyses, according to the analytical standards of the Adolfo Lutz Institute [82]:

- pH: determined in 10 mL of the concentrated juice with a pH meter (Hanna, Smithfield, RI, USA, model pH 21).
- Soluble solids (SS): determined with three drops of the concentrated juice using a digital refractometer with automatic temperature compensation (Asko, Oslo, Norway, model RHB32), previously calibrated with deionized water. The quantification was performed in triplicate and the average data expressed in °Brix.
- Titratable acidity (TA): determined by potentiometric volumetry, where the concentrated juice (10 mL) was diluted in 100 mL of deionized water and this mixture was titrated with 0.1 N NaOH solution until reaching pH of 8.2. The results were expressed as % citric acid.
- SS/TA ratio: maturity index obtained by the soluble solids to titratable acidity ratio.

The other half of each sample (30 fruits), still frozen, was macerated in liquid nitrogen until a fine powder was obtained, which was used for the following analyses, in triplicate:

- Soluble sugars: extraction was performed according to methodology proposed by Garcia et al. [83], where 100 mg of the plant material was dissolved in 1 mL of 80% ethanol; the mixture was incubated in water bath at 80 °C for 15 min and centrifuged at 12,000 rpm at 25 °C for 15 min. The plant material was submitted to three extractions and the supernatants were combined and equalized with deionized water to a final volume of 3 mL. Quantification was performed by the phenol-sulfuric method [84], where a 10 µL aliquot of the extract was incorporated into 490 µL of deionized water, 0.5 mL of 5% phenol and 2.5 mL of concentrated sulfuric acid. The solution was homogenized, cooled to room temperature for 5 min and read on a UV-Vis spectrophotometer (Bel Engineering^®^, Monza, Italy, model UV-M51) at 490 nm. The concentration of soluble sugars was calculated using an anhydrous glucose calibration curve (y = 0.0183x + 0.0719, R^2^ = 0.9998) and expressed in milligrams of glucose equivalent per gram of fresh mass.
- Total phenols: equivalent to the concentration of phenolic compounds in the sample, quantified according to the Folin-Ciocalteau method, with adaptations [85]. The plant material (100 mg) was dissolved in 5 mL of 50% acetone and the mixture was vortexed for 30 s, submitted to ultrasonic bath for 20 s and centrifuged at 5000 rpm for 10 min. Two extractions were performed and the supernatants were combined. An aliquot of 0.5 mL of the extract was incorporated into 0.5 mL of deionized water, 0.5 mL of Folin-Ciocalteau reagent (1:4) and 2.5 mL of 4% Na_2_CO_3_. After homogenization and remaining in the dark and at room temperature for one hour, reading was performed on a UV-Vis spectrophotometer at 725 nm. The concentration of total phenols was calculated using the gallic acid calibration curve (y = 0.0265x + 0.0032, R^2^ = 0.9955) and expressed in milligrams of gallic acid equivalent per gram of fresh mass.
- Flavonoids: the plant material (100 mg) was dissolved in 4 mL of acidified methanol (85:15, 70% methanol: 10% acetic acid); the mixture was vortexed for 30 s, submitted to ultrasonic bath for 30 min and subsequently incorporated into 1 mL of 5% aluminum chloride. After homogenization and remaining in the dark and at room temperature for 30 min, centrifugation was performed at 7830 rpm at 5 °C for 22 min and the extracted supernatant was read in a UV-Vis spectrophotometer at 425 nm [86,87]. The concentration of total flavonoids was determined by the rutin calibration curve (y = 0.0026x − 0.0009, R^2^ = 0.9999) and the results were expressed in milligrams of rutin equivalent per gram of fresh mass.
- Anthocyanins: determination by differential pH method, with adaptations [88]. For extraction, 300 mg of plant material was diluted in 13 mL of extracting solution (99:1; MeOH: 1 N HCl), remaining at rest for 24 h at 4 °C. After this period, the mixture was sonicated for 60 min, centrifuged at 5000 rpm for 10 min and, finally, the supernatant was separated from the plant material. The extract was dissolved separately in two buffer solutions, one at pH 1.0 (KCl, 0.025 M) and the other at pH 4.5 (CH_3_COONa, 0.40 M), in the proportion of 1.0 mL of extract to 3.0 mL of each solution. The absorbance of each dilution was measured in a UV-Vis spectrophotometer at 520 and 700 nm, using the extracting solution as a blank. Quantification was performed using the following formula: anthocyanin pigment (mg/mL) = A × MW × DF/(ε × I). Where A = (A520 nm − A700 nm) pH 1.0 − (A520 nm − A700 nm) pH 4.5; MW (molecular weight) = 449.2 g/mol of cyanidin-3-glucoside; DF = dilution factor; ε (extinction coefficient, in mol/L of cyanidin-3-glucoside) = 26,900 molar; I = cuvette thickness in cm (1.0). Results were expressed in milligram equivalents of cyanidin-3-glucoside per gram of fresh mass.
- Antioxidant activity: determination according to the method that evaluates the scavenging capacity of 2,2-diphenyl-1-picrylhydrazyl (DPPH) radicals, with adaptations [89,90]. The plant material (200 mg) was dissolved in 5 mL of acidified methanol (80:19:1, methanol:deionized water:acetic acid). This mixture was vortexed for 10 s, submitted to ultrasonic bath for 15 min and centrifuged at 2000 rpm at 5 °C for 10 min, and the supernatant was subsequently extracted. An aliquot of 500 μL of the supernatant was incorporated into 3 mL of concentrated ethanol and 300 μL of the DPPH solution (2 × 10^−4^ g mL^−1^). After homogenization and remaining in the dark and at room temperature for 40 min, reading was performed, together with the blank, in a UV-Vis spectrophotometer at 517 nm. Results were expressed in % of reduced DPPH, using the following formula: % reduced DPPH = (Blank Absorbance − Sample Absorbance)/Blank Absorbance × 100.

Based on the statistical analysis of the chemical characteristics and antioxidant composition of fruits in the 12 treatments, five treatments with plant growth regulators and control were selected for analysis on two other dates (beginning and end of the yield cycle), aiming to evaluate the intensity of the effect of treatments throughout the cycle. At the end of the yield cycle, samples consisted of 60 fruits, while at the beginning of the yield cycle, they consisted of 30 fruits, due to lower availability.

### 4.5. Data Analysis

Data normality was verified by the Shapiro-Wilk test and homogeneity was confirmed by the Levene test. Subsequently, a one-way analysis of variance and the Scott-Knott multiple comparison test at 5% probability were performed to verify whether there were significant differences between treatments. The statistical analyses were performed using the Sisvar v. 5.8 software.

## 5. Conclusions

Plant growth regulators GA_3_ and BA, alone and combined in different concentrations, impact the chemical characteristics and antioxidant composition of fruits, especially at the beginning of the yield cycle and at peak yield, 28 and 70 days after the start of supplying GA_3_ and BA, respectively. However, plant growth regulators do not alter the productive and physical characteristics of blueberry ‘Biloxi’ fruits in the analyzed cycle.

The increases in the antioxidant activity and concentrations of specialized metabolites (total phenols, flavonoids and anthocyanins), soluble sugars, SS and SS/TA ratio in fruits are more evident when GA_3_ and BA are used in combination at their highest concentrations (100 mg L^−1^), resulting in fruits with greater nutraceutical and sensory quality.

It could also be concluded that the exogenous GA_3_ and BA application can be considered a promising practice for obtaining blueberries with higher quality.

## Figures and Tables

**Table 1:** Effect of gibberellic acid (GA_3_) and benzylaminopurine (BA) on the concentrations of total phenols, flavonoids, anthocyanins and on the antioxidant activity of *Vaccinium corymbosum* ‘Biloxi’ fruits at peak yield, 70 days after the start of applications (DASA).

Treatments	Total Phenols (mg g^−1^)	Flavonoids (mg g^−1^)	Anthocyanins (mg g^−1^)	Antioxidant Activity (%)
Control	2.95 ± 0.09 b	2.00 ± 0.03 b	1.35 ± 0.01 b	89.64 ± 0.28 b
GA25	3.16 ± 0.12 b	1.96 ± 0.03 b	1.31 ± 0.04 b	88.17 ± 0.26 c
GA50	2.83 ± 0.13 b	**2.16 ± 0.08 a**	1.26 ± 0.06 b	**89.91 ± 0.45 a**
GA100	3.09 ± 0.16 b	1.85 ± 0.06 b	**1.47 ± 0.05 a**	**90.52 ± 0.21 a**
BA50	3.08 ± 0.12 b	**2.12 ± 0.06 a**	**1.51 ± 0.03 a**	89.04 ± 0.26 b
BA100	3.05 ± 0.12 b	**2.18 ± 0.03 a**	1.35 ± 0.05 b	87.72 ± 0.35 c
GA25 + BA50	2.79 ± 0.13 b	1.90 ± 0.08 b	**1.51 ± 0.07 a**	87.52 ± 0350 c
GA25 + BA100	**3.39 ± 0.13 a**	2.02 ± 0.04 b	**1.45 ± 0.02 a**	89.08 ± 0.13 b
GA50 + BA50	3.16 ± 0.20 b	1.87 ± 0.06 b	1.32 ± 0.05 b	89.35 ± 0.10 b
GA50 + BA100	**3.51 ± 0.11 a**	2.01 ± 0.02 b	**1.47 ± 0.04 a**	**90.35 ± 0.11 a**
GA100 + BA50	**3.48 ± 0.06 a**	2.00 ± 0.10 b	**1.43 ± 0.09 a**	**90.28 ± 0.58 a**
GA100 + BA100	**3.52 ± 0.13 a**	**2.09 ± 0.01 a**	**1.47 ± 0.03 a**	**90.97 ± 0.16 a**
*p*	0.0167 *	0.0053 ^n.s.^	0.0317 *	<0.0001 *
F	2.47	2.94	2.20	10.90
CV (%)	10.84	7.04	9.11	0.85

Results are presented as the mean value ± the standard deviation. Values were statistically tested using one-way ANOVA. Means followed by the same letters do not differ by the Scott-Knott test at 5% probability. *p*, F and coefficient of variation (CV) values (ANOVA) are indicated. ^n.s.^: not significant (*p*-value > 0.05); * 5% level of significance (*p*-value ≤ 0.05); bold font is used to highlight the means of greatest statistical significance.

**Table 2:** Effect of gibberellic acid (GA_3_) and benzylaminopurine (BA) on the concentrations of total phenols, flavonoids, anthocyanins and antioxidant activity of *Vaccinium corymbosum* ‘Biloxi’ fruits at the beginning and end of the 2023 yield cycle, 28 days after the start of application (DASA).

Beginning of the Yield Cycle (28 DASA)				
Treatments	Total Phenols (mg g^−1^)	Flavonoids (mg g^−1^)	Anthocyanins (mg g^−1^)	Antioxidant Activity (%)
Control	3.45 ± 0.05 b	2.27 ± 0.02 b	1.19 ± 0.02 b	90.76 ± 0.19 b
GA100	**3.74 ± 0.11 a**	2.48 ± 0.11 b	**1.40 ± 0.06 a**	90.38 ± 0.17 b
BA100	**4.00 ± 0.08 a**	**2.70 ± 0.10 a**	**1.36 ± 0.04 a**	90.78 ± 0.29 b
GA25 + BA50	**3.95 ± 0.09 a**	2.49 ± 0.10 b	**1.35 ± 0.03 a**	89.84 ± 0.29 b
GA100 + BA50	**3.78 ± 0.06 a**	**2.82 ± 0.10 a**	**1.47 ± 0.06 a**	90.33 ± 0.10 b
GA100 + BA100	**3.92 ± 0.03 a**	2.31 ± 0.10 b	**1.34 ± 0.02 a**	**91.94 ± 0.23 a**
*p*	0.0012 *	0.0124 *	0.0180 *	<0.0001 *
F	6.28	3.91	3.57	9.63
CV (%)	4.68	9.65	8.28	0.56

Results are presented as the mean value ± the standard deviation. Values were statistically tested using one-way ANOVA. Means followed by the same letters do not differ by the Scott-Knott test at 5% probability. *p*, F and coefficient of variation (CV) values (ANOVA) are indicated. * 5% level of significance (*p*-value ≤ 0.05); bold font is used to highlight the means of greatest statistical significance.

**Table 3:** Effect of gibberellic acid (GA_3_) and benzylaminopurine (BA) on the concentrations of soluble sugars, soluble solids (SS), titratable acidity (TA), SS/TA ratio and pH of *Vaccinium corymbosum* ‘Biloxi’ fruits at peak yield, 70 days after the start of applications (DASA).

Treatments	Soluble Sugars (mg g^−1^)	SS (°Brix)	TA (% C.A.)	SS/TA Ratio	pH
Control	75.88 ± 2.09 b	10.20 ± 0.18 b	0.64 ± 0.01 b	**16.06 ± 0.26 a**	3.22 ± 0.03 a
GA25	68.85 ± 3.14 b	10.05 ± 0.07 b	0.71 ± 0.02 b	14.22 ± 0.45 b	3.19 ± 0.02 a
GA50	70.39 ± 2.43 b	9.80 ± 0.18 b	0.70 ± 0.03 b	14.13 ± 0.80 b	3.22 ± 0.02 a
GA100	**81.50 ± 0.69 a**	**10.35 ± 0.10 a**	0.67 ± 0.02 b	**15.41 ± 0.42 a**	3.23 ± 0.03 a
BA50	70.35 ± 2.07 b	9.75 ± 0.18 b	**0.75 ± 0.02 a**	12.97 ± 0.40 b	3.21 ± 0.02 a
BA100	**78.84 ± 2.28 a**	**10.85 ± 0.18 a**	0.68 ± 0.02 b	**15.95 ± 0.49 a**	3.22 ± 0.01 a
GA25 + BA50	**81.36 ± 1.98 a**	9.95 ± 0.03 b	0.63 ± 0.03 b	**15.86 ± 0.73 a**	3.22 ± 0.03 a
GA25 + BA100	**86.14 ± 1.85 a**	9.95 ± 0.03 b	**0.76 ± 0.02 a**	13.13 ± 0.37 b	3.19 ± 0.02 a
GA50 + BA50	**83.88 ± 2.93 a**	**10** **.73 ± 0.10 a**	0.65 ± 0.02 b	**16.65 ± 0.66 a**	3.25 ± 0.02 a
GA50 + BA100	**82.92 ± 1.81 a**	**10.45 ± 0.14 a**	**0.77 ± 0.03 a**	13.65 ± 0.62 b	3.17 ± 0.02 a
GA100 + BA50	**78.53 ± 1.97 a**	**10.85 ± 0.03 a**	**0.77 ± 0.01 a**	14.03 ± 0.22 b	3.16 ± 0.01 a
GA100 + BA100	**83.90 ± 2.27 a**	**10.63 ± 0.17 a**	0.71 ± 0.00 b	**15.05 ± 0.31 a**	3.22 ± 0.01 a
*p*	<0.0001 *	<0.0001 *	0.0001 *	0.0002 *	0.1952 ^n.s.^
F	5.52	7.09	4.52	4.31	1.43
CV (%)	7.18	3.29	7.71	8.99	1.47

Results are presented as the mean value ± the standard deviation. Values were statistically tested using one-way ANOVA. Means followed by the same letters do not differ by the Scott-Knott test at 5% probability. *p*, F and coefficient of variation (CV) values (ANOVA) are indicated. ^n.s.^: not significant (*p*-value > 0.05); * 5% level of significance (*p*-value ≤ 0.05); bold font is used to highlight the means of greatest statistical significance.

**Table 4:** Effect of gibberellic acid (GA_3_) and benzylaminopurine (BA) on the concentrations of soluble sugars, soluble solids (SS), titratable acidity (TA), SS/TA ratio and pH of *Vaccinium corymbosum* ‘Biloxi’ fruits at the beginning and end of the 2023 yield cycle, 28 days after the start of application (DASA).

	Beginning of the Yield Cycle (28 DASA)				
Treatments	Soluble Sugars (mg g^−1^)	SS (°Brix)	TA (% C.A.)	SS/TA Ratio	pH
Control	103.49 ± 0.94 b	13.04 ± 0.15 b	1.17 ± 0.03 b	11.15 ± 0.35 b	2.78 ± 0.02 a
GA100	**113.56 ± 1.40 a**	13.00 ± 0.37 b	**1.22 ± 0.02 a**	10.64 ± 0.31 b	2.79 ± 0.02 a
BA100	**115.58 ± 3.66 a**	**14.08 ± 0.20 a**	**1.26 ± 0.04 a**	11.25 ± 0.39 b	2.81 ± 0.02 a
GA25 + BA50	**121.89 ± 3.80 a**	12.44 ± 0.36 b	**1.27 ± 0.04 a**	9.81 ± 0.41 b	2.73 ± 0.03 a
GA100 + BA50	**115.07 ± 2.66 a**	**13.56 ± 0.30 a**	**1.28 ± 0.03 a**	10.68 ± 0.51 b	2.79 ± 0.04 a
GA100 + BA100	**115.23 ± 2.72 a**	**13.56 ± 0.13 a**	1.08 ± 0.02 b	**12.61 ± 0.26 a**	2.86 ± 0.03 a
*p*	0.0043 *	0.0135 *	0.0053 *	0.0102 *	0.1381 ^n.s.^
F	4.91	3.83	4.71	4.08	1.91
CV (%)	5.27	4.92	6.58	9.35	2.38

Results are presented as the mean value ± the standard deviation. Values were statistically tested using one-way ANOVA. Means followed by the same letters do not differ by the Scott-Knott test at 5% probability. *p*, F and coefficient of variation (CV) values (ANOVA) are indicated. ^n.s.^: not significant (*p*-value > 0.05); * 5% level of significance (*p*-value ≤ 0.05); bold font is used to highlight the means of greatest statistical significance.

**Table 5:** Average temperature, relative air humidity and rainfall during the experiment with *Vaccinium corymbosum* ‘Biloxi’ fruits in 2023.

Months	Average Temperature (°C)	Average Relative Air Humidity (%)	Rainfall (mm)
July	19.76	69.64	0.00
August	23.08	60.76	13.50
September	26.65	58.29	37.00
October	26.11	73.21	221.30
November	27.47	66.25	72.50
Average	24.61	65.63	-
Total rainfall	-	-	344.30

**Table 6:** Gibberellic acid (GA_3_) and benzylaminopurine (BA) treatments exogenously supplied to *Vaccinium corymbosum* ‘Biloxi’ plants.

Treatment	GA_3_ (mg L^−1^)	BA (mg L^−1^)	Identification
T1 *	-	-	Control
T2	25	-	GA25
T3	50	-	GA50
T4	100	-	GA100
T5	-	50	BA50
T6	-	100	BA100
T7	25	50	GA25 + BA50
T8	25	100	GA25 + BA100
T9	50	50	GA50 + BA50
T10	50	100	GA50 + BA100
T11	100	50	GA100 + BA50
T12	100	100	GA100 + BA100

* Only water and Silwet^®^ surfactant at concentration of 0.05%.

## Data Availability

Data are contained in the article and Supplementary Materials.

## Supplementary Materials

The following supporting information can be downloaded at: https://www.mdpi.com/article/10.3390/ijms26167984/s1.
