# Supplementary material for: Exogenous Gibberellic Acid (GA3) and Benzylaminopurine Enhance the Antioxidant Properties of Vaccinium corymbosum L. ‘Biloxi’ Fruits Without Affecting Yield
Source: Int J Mol Sci. 2025 Aug 19;26(16):7984. doi: 10.3390/ijms26167984 (PMC12386784; doi:10.3390/ijms26167984)
Supplement: Supplementary file 1 [file ijms-26-07984-s001.zip › ijms-3716456-supplementary.pdf]

Supplementary Materials

Table S1. Summary of ANOVA for yield (YD), number of fruits (NF), fruit mass (FM), fruit equatorial diameter (FED) and fruit polar diameter (FPD) in *Vaccinium corymbosum* 'Biloxi' submitted to exogenous gibberellic acid (GA_3) and benzylaminopurine (BA) days after the start of application (DASA) in the 2023 yield cycle.

DASA	YD (g plant^{-1})		NF (no. planta^{-1})		FM (g)		FED (mm)		FPD (mm)	
	p	CV	*p*	CV	*p*	CV	*p*	CV	*p*	CV
21	0.3525	72.53	0.6554	78.24	0.5167	10.40	0.9165	4.08	0.5284	5.32
28	0.7676	84.12	0.7740	85.07	0.4996	11.74	0.2384	4.70	0.3828	4.80
35	0.7282	90.00	0.6856	85.96	0.9438	11.67	0.9369	5.09	0.9915	5.26
42	0.6031	63.89	0.8981	54.81	0.6773	10.52	0.7745	3.52	0.4038	3.76
49	0.7706	48.11	0.7763	48.12	0.2373	8.67	0.2935	3.33	0.6242	2.91
56	0.8915	31.02	0.8660	33.54	0.2703	7.12	0.0527	2.73	0.5379	2.80
63	0.9001	32.12	0.7632	34.47	0.3544	7.34	0.2791	2.43	0.2871	2.05
70	0.5217	28.82	0.4643	31.60	0.3819	8.18	0.1188	3.09	0.4537	2.36
77	0.2866	26.93	0.5375	28.62	0.5315	8.17	0.6568	3.70	0.7924	2.81
84	0.3792	25.32	0.4437	29.11	0.4608	9.02	0.4754	4.67	0.5197	3.85
91	0.4855	39.18	0.3806	39.67	0.6573	9.68	0.9699	4.74	0.9882	4.39
98	0.0927	35.47	0.1154	35.65	0.6953	8.99	0.2854	3.84	0.0540	3.33
105	0.0519	36.62	0.0782	33.84	0.3155	10.22	0.1833	2.59	0.0710	2.79
112	0.5332	44.42	0.3108	39.58	0.9058	11.41	0.9422	3.74	0.08	3.27

Coefficient of variation in % (CV).

Table S2. Effect of gibberellic acid (GA_3) and benzylaminopurine (BA) on average yield (AYD - g plant^{-1}), average number of fruits (ANF - № plant^{-1}), average fruits mass (AFM - g), average fruit equatorial diameter (AFED - mm) and average fruit polar diameter (AFPD - mm), in *Vaccinium corymbosum* 'Biloxi', 2023 production cycle, from 21 to 112 days after start of applications (DASA).

Treatments	AYD (g plant^{-1})	ANF (№ plant^{-1})	AFM (g)	AFED (mm)	AFPD (mm)
Control	924.19 ± 49.11 a	765.00 ± 28.21 a	1.30 ± 0.03 a	14.04 ± 0.13 a	10.45 ± 0.07 a
GA25	984.15 ± 90.84 a	786.33 ± 72.25 a	1.31 ± 0.03 a	14.19 ± 0.12 a	10.54 ± 0.08 a
GA50	1029.40 ± 43.76 a	893.73 ± 70.91 a	1.32 ± 0.05 a	14.12 ± 0.20 a	10.51 ± 0.12 a
GA100	1010.27 ± 88.24 a	862.40 ± 87.75 a	1.28 ± 0.05 a	14.06 ± 0.13 a	10.52 ± 0.11 a
BA50	929.96 ± 40.08 a	749.67 ± 43.25 a	1.33 ± 0.03 a	14.23 ± 0.12 a	10.55 ± 0.08 a
BA100	940.94 ± 107.57 a	801.73 ± 92.83 a	1.26 ± 0.05 a	13.85 ± 0.20 a	10.40 ± 0.13 a
GA25+BA50	981.06 ± 91.30 a	799.53 ± 74.54 a	1.36 ± 0.04 a	14.25 ± 0.14 a	10.61 ± 0.10 a
GA25+BA100	958.82 ± 73.18 a	793.00 ± 54.65 a	1.32 ± 0.05 a	14.11 ± 0.19 a	10.57 ± 0.13 a
GA50+BA50	898.44 ± 43.79 a	725.80 ± 31.37 a	1.35 ± 0.02 a	14.28 ± 0.08 a	10.62 ± 0.08 a
GA50+BA100	958.67 ± 39.75 a	817.40 ± 25.89 a	1.26 ± 0.05 a	13.90 ± 0.19 a	10.41 ± 0.11 a
GA100+BA50	981.24 ± 68.29 a	814.80 ± 67.11 a	1.29 ± 0.03 a	14.10 ± 0.12 a	10.50 ± 0.07 a
GA100+BA100	1005.27 ± 55.25 a	827.27 ± 47.32 a	1.29 ± 0.03 a	13.98 ± 0.10 a	10.52 ± 0.11 a
p	0.9928 $^{n.s.}$	0.9430 $^{n.s.}$	0.8478 $^{n.s.}$	0.7244 $^{n.s.}$	0.9352 $^{n.s.}$
F	0.24	0.41	0.56	0.71	0.43

CV (%)	18.32	19.98	7.13	2.52	2.22

Results are presented as the mean value ± the standard deviation. Values were statistically tested using one-way ANOVA. Means followed by the same letters do not differ by the Scott-Knott test at 5% probability. *p*, F and coefficient of variation (CV) values (ANOVA) are indicated. [n.s.]: not significant (p-value > 0.05).

Table S3. Effect of gibberellic acid (GA_3) and benzylaminopurine (BA) on the concentrations of total phenols, flavonoids, anthocyanins and antioxidant activity of *Vaccinium corymbosum* 'Biloxi' fruits at the beginning and end of the 2023 yield cycle, 112 days after the start of application (DASA).

End of the yield cycle (112 DASA)				
**Treatments**	**Total Phenols (mg g^{-1})**	**Flavonoids (mg g^{-1})**	**Anthocyanins (mg g^{-1})**	**Antioxidant activity (%)**
Control	4.44 ± 0.13 a	3.40 ± 0.12 a	1.96 ± 0.06 a	85.89 ± 0.81 a
GA100	4.52 ± 0.07 a	3.27 ± 0.13 a	1.84 ± 0.03 a	87.75 ± 0.37 a
BA100	4.53 ± 0.05 a	3.47 ± 0.13 a	2.01 ± 0.07 a	87.15 ± 0.59 a
GA25+BA50	4.45 ± 0.02 a	2.97 ± 0.05 a	1.93 ± 0.05 a	88.55 ± 0.47 a
GA100+BA50	4.23 ± 0.07 a	3.22 ± 0.13 a	2.02 ± 0.02 a	86.53 ± 1.03 a
GA100+BA100	4.22 ± 0.09 a	3.27 ± 0.06 a	2.03 ± 0.02 a	86.72 ± 0.31 a
p	0.0896 [n.s.]	0.1484 [n.s.]	0.1732 [n.s.]	0.0565 [n.s.]
F	2.24	1.85	1.73	2.61
CV (%)	4.70	8.60	6.19	1.35

Results are presented as the mean value ± the standard deviation. Values were statistically tested using one-way ANOVA. Means followed by the same letters do not differ by the Scott-Knott test at 5% probability. *p*, F and coefficient of variation (CV) values (ANOVA) are indicated. [n.s.]: not significant (*p*-value > 0.05); * 5% of significance (*p*-value ≤ 0.05).

Table S4. Effect of gibberellic acid (GA_3) and benzylaminopurine (BA) on the concentrations of soluble sugars, soluble solids (SS), titratable acidity (TA), SS/TA ratio and pH of *Vaccinium corymbosum* 'Biloxi' fruits at the beginning and end of the 2023 yield cycle, 112 days after the start of application (DASA).

End of the yield cycle (112 DASA)					
**Treatments**	**Soluble sugars (mg g^{-1})**	**SS (°Brix)**	**TA (% C.A.)**	**SS/TA ratio**	**pH**
Control	137.52 ± 1.31 b	15.48 ± 0.13 b	0.68 ± 0.03 a	22.91 ± 1.27 a	3.27 ± 0.08 a
GA100	135.03 ± 5.93 b	15.60 ± 0.15 b	0.66 ± 0.01 a	23.67 ± 0.59 a	3.40 ± 0.03 a
BA100	131.45 ± 4.39 b	15.35 ± 0.03 b	0.72 ± 0.02 a	21.49 ± 0.72 a	3.27 ± 0.06 a
GA25+BA50	126.23 ± 2.56 b	15.05 ± 0.09 b	0.61 ± 0.02 a	24.73 ± 0.89 a	3.42 ± 0.05 a
GA100+BA50	**156.85 ± 4.38 a**	**16.00 ± 0.15 a**	0.66 ± 0.03 a	24.34 ± 1.09 a	3.33 ± 0.06 a
GA100+BA100	139.77 ± 1.14 b	15.40 ± 0.10 b	0.71 ± 0.02 a	21.89 ± 0.70 a	3.31 ± 0.04 a
p	0.0029 *	0.0034 *	0.1332 [n.s.]	0.1895 [n.s.]	0.2641 [n.s.]
F	5.31	5.14	1.94	1.66	1.41
CV (%)	7.37	2.00	9.07	9.79	3.68

Results are presented as the mean value ± the standard deviation. Values were statistically tested using one-way ANOVA. Means followed by the same letters do not differ by the Scott-Knott test at 5% probability. *p*,

F and coefficient of variation (CV) values (ANOVA) are indicated. [n.s.]: not significant (p-value > 0.05); * 5% of significance (p-value ≤ 0.05); bold font was used to highlight the means of greatest statistical significance.
